# EHMTI-0327. Information and communication technology for improving the management of medication overuse headache: results of the comoestas multicentric, multinational study

**DOI:** 10.1186/1129-2377-15-S1-D63

**Published:** 2014-09-18

**Authors:** C Tassorelli, RH Jensen, R De Icco, M Allena, Z Katzarava, JM Lainez, JA Leston, R Fadic, S Spadafora, M Pagani, N Giuseppe

**Affiliations:** 1 Headache Science Centre Dept. of Brain and Behavioural Sciences, C. Mondino National Neurological Institute and University of Pavia, Pavia, Italy; 2 Danish Headache Centre Dept. of Neurology, Glostrup Hospital Faculty of Health Sciences University of Copenhagen, Copenhagen, Denmark; 3 Headache Science Centre Dept of Brain and Behavioural Sciences, C. Mondino National Neurological Institute and University of Pavia, Pavia, Italy; 4 Headache Science Centre, C. Mondino National Neurological Institute, Pavia, Italy; 5 Dept. of Neurology, University of Essen, Essen, Germany; 6 Foundation of the Valencian Community, University Clinical Hospital, Valencia, Spain; 7 Headache Centre, Foundation for Combating Neurological Diseases of Childhood, Buenos Aires, Argentina; 8 Dept. of Neurology, Pontificia Catolica University of Chile, Santiago, Chile; 9 Medical Informatics, ISalud University, Buenos Aires, Argentina; 10 Directorate, Bioengeneering and Medical Informatics Consortium, Pavia, Italy

## Background

The management of medication overuse headache (MOH) is rewarding but also challenging because effective treatment is frequently followed by relapses. Information and Communication Technology has recently been proposed as a valid aid to improve healthcare quality in chronic conditions. In this frame, we developed a headache diary associated with an alert/alarm logic for the interactive monitoring and the professional support of MOH patients after detoxification.

## Aim

Assessment of the acceptability and the effectiveness of this interactive monitoring system, in association with pharmacological treatment, on the outcome of MOH subjects.

## Methods

A parallel-arm, single-blind study was conducted in 4 European Countries and 2 Latin-American Countries. A total of 499 subjects completed the study. Both Groups of patients underwent detoxification and were prescribed prophylactic medications (if required) at baseline. Subsequently, subjects in Group 1 were followed-up with a paper diary and periodical visits for a period of 6 months, while subjects in Group 2 were followed-up with the interactive monitoring system for the same length of time.

## Results

We observed a highly significant improvement of most outcome parameters in both Groups. However, subjects in Group 2 presented a significantly higher percentage of cured cases (92.4% vs 83.3%, p<0.001), a lower rate of relapsers (6.2% vs 9.1%, p<0.003) and an improved patient satisfaction (8.4+1.7 vs 6.9+1.9, p<0.05).

## Conclusions

Adoption of an interactive monitoring system is well accepted by MOH patients and it is associated to a more favorable outcome after detoxification. (EC FP7, contract number 215366)

No conflict of interest.

